# Variants of CEP68 Gene Are Associated with Acute Urticaria/Angioedema Induced by Multiple Non-Steroidal Anti-Inflammatory Drugs

**DOI:** 10.1371/journal.pone.0090966

**Published:** 2014-03-11

**Authors:** José Antonio Cornejo-García, Carlos Flores, María C. Plaza-Serón, Marialbert Acosta-Herrera, Natalia Blanca-López, Inmaculada Doña, María J. Torres, Cristobalina Mayorga, Rosa M. Guéant-Rodríguez, Pedro Ayuso, Javier Fernández, José J. Laguna, José A. G. Agúndez, Elena García-Martín, Jean-Louis Guéant, Gabriela Canto, Miguel Blanca

**Affiliations:** 1 Research Laboratory, IBIMA-Málaga University General Hospital, Málaga, Spain; 2 Centro de Investigación Biomédica en Red (CIBER) de Enfermedades Respiratorias, Instituto de Salud Carlos III, Madrid, Spain; 3 Research Unit, Hospital Universitario N.S. de Candelaria, Tenerife, Spain; 4 Applied Genomics Group (G2A), Genetics Laboratory, Instituto Universitario de Enfermedades Tropicales y Salud Pública de Canarias, Universidad de La Laguna, Tenerife, Spain; 5 Research Unit, Hospital Universitario Dr. Negrín, Las Palmas de Gran Canaria, Spain; 6 Allergy Service, Infanta Leonor Hospital, Madrid, Spain; 7 Unidad de Gestión Clinica (UGC) Allergy-Instituto de Investigación Biomédica (IBIMA), Málaga University General Hospital, Málaga, Spain; 8 Institut National de la Santé et de la Recherche Médicale (INSERM) U-954, Nutrition-Génétique et exposition aux risques environmentaux, Faculty of Medicine, University of Nancy, Vandoeuvre-les-Nancy, France; 9 University Hospital Center (CHU) of Nancy, Vandoeuvre-les-Nancy, France; 10 Allergy Service, Alicante Hospital, Alicante, Spain; 11 Allergy Service, Cruz Roja Central Hospital, Madrid, Spain; 12 Pharmacology Department, University of Extremadura, Cáceres, Spain; 13 Biochemistry, Molecular Biology and Genetics Department, University of Extremadura, Cáceres, Spain; Kunming Institute of Zoology, Chinese Academy of Sciences, China

## Abstract

Non-steroidal anti-inflammatory drugs (NSAIDs) are the most consumed drugs worldwide because of their efficacy and utility in the treatment of pain and inflammatory diseases. However, they are also responsible for an important number of adverse effects including hypersensitivity reactions. The most important group of these reactions is triggered by non-immunological, pharmacological mechanisms catalogued under the denomination of cross-intolerance (CRI), with acute urticaria/angioedema induced by multiple NSAIDs (MNSAID-UA) the most frequently associated clinical entity. A recent genome-wide association study identified the gene encoding the centrosomal protein of 68 KDa (*CEP68*) as the major locus associated with aspirin intolerance susceptibility in asthmatics. In this study, we aimed to assess the role of this locus in susceptibility to CRI to NSAIDs by examining 53 common gene variants in a total of 635 patients that were classified as MNSAID-UA (n = 399), airway exacerbations (n = 110) or blended pattern (n = 126), and 425 controls. We found in the MNSAID-UA group a number of variants (17) associated (lowest *p*-value = 1.13×10^−6^), including the non-synonymous Gly74Ser variant (rs7572857) previously associated with aspirin intolerance susceptibility in asthmatics. Although not being significant in the context of multiple testing, eight of these variants were also associated with exacerbated respiratory disease or blended reactions. Our results suggest that *CEP68* gene variants may play an important role in MNSAID-UA susceptibility and, despite the different regulatory mechanisms involved depending on the specific affected organ, in the development of hypersensitivity reactions to NSAIDs.

## Introduction

Non-steroidal anti-inflammatory drugs (NSAIDs) are the most consumed medicines worldwide because of their efficacy and utility for the treatment of different inflammatory diseases as well as pain [1], [2]. However, they are associated with a broad range of adverse events [3] including hypersensitivity reactions (HRs) [4], [5]. The most important group of HRs to NSAIDs in both adults [5], [6] and children [7] is cross-intolerance (CRI), which is triggered by chemically unrelated drugs, presumably by pharmacological mechanisms [8], [9].

Clinical symptoms of CRI can affect the airways (rhinosinusitis/nasal polyposis and/or asthma), a condition known as aspirin-induced asthma or aspirin-exacerbated respiratory disease (AERD), and the skin (urticaria and/or angioedema, UA) [10]. A mixed pattern involving both systems has also been described (blended reactions) [10]. Multiple NSAIDs-induced UA (MNSAID-UA), i.e. acute UA induced by various NSAIDs in otherwise healthy subjects without history of underlying chronic skin and/or respiratory disease [10] is the most frequent clinical entity induced by CRI [5]–[7]. However, up to now MNSAID-UA has received little attention compared to CRI reactions involving respiratory airways.

The anti-inflammatory actions of NSAIDs are carried out through the inhibition of cyclooxygenase-1 (COX-1), which diminishes the biosynthesis of prostaglandins (PGs) and deviates the metabolism of arachidonic acid (AA) towards the formation of pro-inflammatory cysteinyl-leukotrienes (LTs) (LTC4, LTD4 and LTE4), thus triggering a hypersensitivity response in susceptible individuals. This model was proposed for AERD [11], and has been supported by the increase in the concentration of LTs after aspirin challenge [12]–[16]. As patients with NSAIDs-exacerbated chronic urticaria (CU) showed a similar profile, this hypothesis was also extended to MNSAID-UA [17], [18]. Nevertheless, the inhibition of COX-1 cannot explain either the high basal concentration of LTE4 in urine [19] or the overproduction of PGD2 during bronchoconstriction in AERD [20]. Moreover, a recent study found no differences in the levels of PGE2 [21] and LTs [22], [23] between AERD and asthmatic patients with good tolerance to aspirin.

Apart from the characterization of intermediate phenotypes, considerable efforts have been taken to disentangle the genetics of CRI, mainly through the candidate gene approach. Most studies have considered AERD or CU [24]–[30], however MNSAID-UA is now being analyzed in more detail [31], [32]. Although only two genome-wide association studies (GWAS) have been conducted in CRI, both focusing on AERD [33], [34], this information can be of utility to analyze the underlying mechanisms in MNSAID-UA. The more recent of the two studies suggests a potential role for the HLA system, but the presentation of the parental drug or their metabolites are not thought to be involved in this pathology. Importantly, one of them proposed the *CEP68* gene, encoding the centrosomal protein of 68 kDa, as a susceptibility locus for AERD [33]. In this study we aimed to analyze the potential role of common genetic variants in *CEP68* gene in the predisposition to MNSAID-UA, the most frequent clinical entity in HRs to drugs. We studied a well-characterized group of Spanish patients with MNSAID-UA, defined as skin reactions in the absence of airway exacerbations or underlying chronic urticaria. We also extended this analysis to two small groups of patients with airway exacerbations or with blended reactions. To our knowledge, this is the first time that genes different from those related to AA metabolism or to inflammatory mediators have been analyzed in the context of MNSAID-UA susceptibility.

## Materials and Methods

### Ethics statement

The study was conducted according to the principles of the Declaration of Helsinki and approved by the Ethics Committee of the Carlos Haya Hospital. Written informed consent was obtained from all the participants involved in the study.

### Subjects

The study included a total of 635 unrelated patients with CRI to NSAIDs and 425 unrelated controls, all of self-reporting Spanish ancestry, recruited between 2007 and 2012 from the Allergy Services of five Spanish public hospitals integrated in the national research network for allergic diseases RIRAAF (Table 1).

**Table 1:** Demographic and clinical data of patients and controls.

Variable	Healhty Controls (n = 425)	Patients (n = 635)	p-value
Mean age (in years) ± SD	41.52±15.74	41.93±15.52	0.563
Female number (%)	263 (61.8)	390 (61.4)	0.892
History of allergic disease	59 (13.9)	261 (41.1)	<0.0001
Clinical group:			
MNSAID-AU	0	399 (62.8)	
Airway exacerbations	0	110 (17.3)	
*Asthma*		66 (60.0)	
*Rhinitis*		16 (14.6)	
*AERD*		7 (6.3)	
Blended pattern		126 (19.8)	
No. of episodes	0	3.21±1.65	
No. of drugs involved	0	2.44±0.74	
Culprit NSAIDs, No. of episodes:			
*Propionic acid derivatives*		691 (33.9)	
*Acetylsalicylic acid*		511 (25.0)	
*Pyrazolones*		389 (19.1)	
*Others*		449 (22.0)	

**Abbreviations**: **AERD**, aspirin-exacerbated respiratory disease; **CI**, confidence intervals; **MNSAID-UA**, multiple NSAIDs-induced urticaria/angioedema; **NSAIDs**, non-steroidal anti-inflammatory drugs; **SD**, standard deviation.

To be included, all patients must have presented a clinical history of episodes with more than 2 different NSAIDs without NSAID-exacerbated CU. Diagnosis was confirmed by oral provocation test in a single-blind procedure as described previously [6]. On the first day, placebo capsules were given at different time intervals. At least 1 week later, increasing doses of acetylsalicylic acid were administered orally at intervals of 90 min up to a total of 2-4 administrations. The procedure was stopped if any cutaneous and/or respiratory symptoms or alterations in vital signs (rhythm modifications, decrease in peak expiratory flow rate or hypotension) appeared in which case patients were evaluated and treated. If no symptoms occurred, the therapeutic dose was achieved by taking a course of two additional days giving acetylsalicylic acid 500 mg every eight hours. According to clinical symptoms, patients were assigned to three different groups: MNSAID-UA, airway exacerbations, or blended pattern (Table 1). These three groups were independently compared with the control group, which comprised individuals without any previous history of HRs to any drug despite usually taking NSAIDs.

Those patients who responded to one single drug and showed good tolerance to strong COX-1 inhibitors were classified as selective and therefore excluded from the study. Considering the potential interaction between food allergy and NSAIDs [31], patients with a clinical history of food allergy or positive IgE antibodies for food allergens, despite having no history of food allergy, were not included in the study.

### DNA isolation, single nucleotide polymorphism selection and genotyping

Genomic DNA was isolated from 3 ml of peripheral blood in citrate-coated tubes using FlexiGene DNA kit (Qiagen, Hilden, Germany). The multiple-marker selection algorithm based on haplotype *r* ^2^ included in TagIT 3.03 software [35] was used to select a common set of 6 tagging single nucleotide polymorphisms (tagSNPs) in *CEP68* as previously described [36], satisfying a haplotype *r^2^*>0.80 by using a SNP-dropping-with-resampling method (Table S1). For this, we forced the inclusion of the non-synonymous polymorphism rs7572857, which was previously associated with AERD by a GWAS in Koreans [33].

Genotyping was conducted using TaqMan® allelic discrimination assays in a 7500 Fast Real-Time PCR System (Applied Biosystems, Foster City, CA, USA), with automated calls generated using the 7500 software 2.0.1 based on discriminating plots with 95% confidence. Duplicate samples and negative controls were included across the plates to ensure genotyping quality. Genotyping was blind to case-control status and completion rates were >99% for all tagSNPs. No significant deviations from Hardy-Weinberg equilibrium were observed either in patients or in healthy controls for the genotypes obtained (Table S1). Thus, all tagSNPs were retained for further analyses.

### Functional protein association networks analysis

In order to identify functional associations between CEP68 and other proteins, the STRING database [37] (available online at http://string-db.org) was used.

### Statistical analysis

The Mann-Whitney U-test and the χ^2^-test were used for the comparison of demographic and clinical variables using SPSS v.15 (SPSS Inc., Chicago, IL, USA). Hardy-Weinberg equilibrium (HWE) assessments for cases and controls were done with an exact test with SNPing [38], [39]. SNP imputation was carried out with MaCH 1.0 [40] using as reference the Phase 1 data for European samples (May 2011) deposited in the 1000 Genomes Project Consortium [41]. Association testing was conducted with Mach2dat [40] using logistic regression for allele dosages. This was done for a total of 53 SNPs (47 imputed and 6 genotyped) showing a MAF≥10% and a squared correlation between imputed and true genotypes (Rsq)>0.3, as provided by MaCH (Table S2). Independence of SNP associations were explored by means of conditional regression analysis using R 2.15.0 (http://www.r-project.org/). Representation of association results was then performed using LocusZoom 1.1 [42] based on linkage disequilibrium (LD) data from hg19 deposited for European population by the 1000 Genomes Project. Predicted functional effects were evaluated according to FASTSNP [43] and SNPinfo [44].

A *p*-value ≤9.4×10^−4^ was considered statistically significant following a Bonferroni correction for the multiple comparisons (0.05/53). As this correction can be conservative, we also considered the associations with a false discovery rate (FDR) using QVALUE for R [45]. However, no correction was applied for the multiple traits compared. Using Power 3.0 [46] we estimate that the study sample size allows to attain >80% power to detect effects >1.85 for variants with MAF = 0.30 at *p* = 9.4×10^−4^.

## Results

The study included a total of 1060 individuals, including 635 patients with CRI to NSAIDs and 425 unrelated, healthy controls, with no significant differences in age or sex between the two groups (*p* = 0.563 and *p* = 0.892, respectively) (Table 1). Although 80% of the patients presented at least 3 episodes, diagnosis for all cases was established by controlled administration of drugs, as described elsewhere [6], [31]. MNSAID-UA was the most frequent clinical entity (62.8%), followed by blended reactions and airway exacerbations (rhinitis and/or asthma) (19.8 and 17.3%, respectively) (Table 1). Propionic acid derivatives were the drugs most frequently involved in these reactions (33.9%), followed by acetylsalicylic acid and pyrazolones (25.0% and 19.1%, respectively) (Table 1).

**Figure 1:**
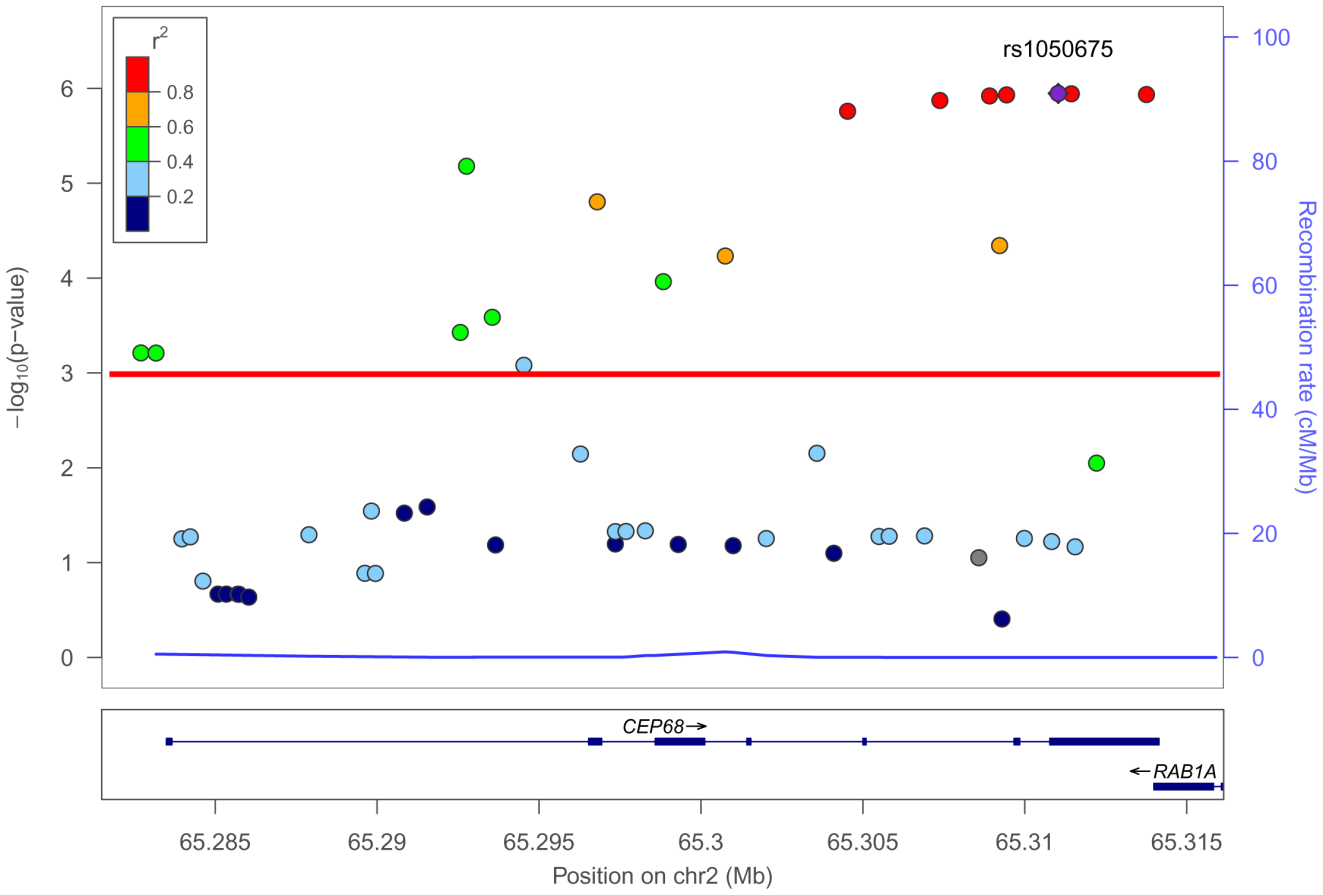
Plot of regional associations for tagging and imputed SNPs in *CEP68* for multiple NSAIDs-induced urticaria/angioedema. The -log_10_p values for association tests are plotted as circles according to their genomic position (NCBI Build 37). The SNP number shown on the plot (indicated by a diamond) denotes the result for the most significant SNP and the results for the remaining were color coded to reflect their LD with this SNP based on pairwise *r* ^2^ values from the European population of the 1000 Genomes Project. Estimated recombination rates (light blue line) were also plotted on the right y-axis to reflect the local LD structure. The horizontal red line indicates a p-value of 9.4×10^−4^.

We found a total of 17 SNPs out of the 53 tested (32%) associated with MNSAID-UA after using both a stringent *p*-value threshold to control type-I error due to multiple testing (Table 2) and an FDR of 5% (Tables S2 and S3, and Figure 1), including the previously associated non-synonymous variant Gly74Ser (rs7572857, OR for Ser [A] allele: 0.55, 95% CI: 0.42–0.73, *p* = 1.67×10^−5^) [33]. The top hit was found at the rs1050675 (OR for G allele: 0.33, 95% CI: 0.21–0.52, *p* = 1.13×10^−6^). However, the results suggested that the association signals of the remaining 16 SNPs were not independent from rs1050675 once its effect was statistically accounted for, as conditioning their association to it, left the remaining non-significant (Table S4).

**Table 2:** Association results and the predicted function for the 17 *CEP68* SNPs surviving the correction for multiple comparisons.

SNPs	Location	Position*	p-value	OR (CI 95%)	Predicted functional effects
rs6728523	5′UTR	65282708	6.15E-04	0.59 (0.43–0.80)	TFBS
rs2302647	5′UTR	65283174	6.17E-04	0.59 (0.43–0.80)	TFBS
rs2901749	Intron	65292570	3.74E-04	0.51 (0.35–0.74)	-
rs2080385	Intron	65292762	6.61E-06	0.43 (0.30–0.62)	TFBS
rs75678687	Intron	65293558	2.59E-04	0.51 (0.35–0.73)	-
rs79157909	Intron	65294532	8.30E-04	0.46 (0.29–0.72)	
rs7572857	Exon 2 (Gly74Ser)	65296798	1.67E-05	0.55 (0.42–0.73)	nsSNP
rs17849707	Exon 2 (Thr203Thr)	65298839	1.09E-04	0.49 (0.34–0.70)	-
rs12621608	Intron	65300752	5.85E-05	0.43 (0.29–0.65)	TFBS
rs76221156	Intron	65304529	1.74E-06	0.34 (0.22–0.53)	-
rs1894874	Intron	65307379	1.34E-06	0.34 (0.22–0.52)	TFBS
rs113359765	Intron	65308913	1.20E-06	0.34 (0.22–0.52)	-
rs6546125	Intron	65309223	4.54E-05	0.44 (0.29–0.65)	-
rs78945874	Intron	65309439	1.17E-06	0.33 (0.22–0.52)	-
rs1050675	3′UTR	65311031	1.13E-06	0.33 (0.21–0.52)	miRNABS
rs1229	3′UTR	65311438	1.14E-06	0.33 (0.21–0.52)	miRNABS
rs61758846	3′UTR	65313758	1.16E-06	0.32 (0.21–0.51)	miRNABS

**Abbreviations**: **miRNABS**, micro RNA binding site; **nsSNP**, non-synonymous single nucleotipe polymorphism; **TFBS**, transcription factor binding site.

* According to NCBI build 37.

Despite the small sample sizes for patients with either airway exacerbations or blended reactions, we also compared them with the control group. In both cases, we found marginal associations for 7 and 8 SNPs, for airway exacerbations and blended reactions, respectively. These SNPs were amongst the 17 SNPs associated with MNSAID-UA, and the lowest *p*-value was obtained for the same SNP (rs61758846; *p* = 0.010 and *p* = 0.013, for airway exacerbations and blended reactions, respectively). However, none of these SNPs remained significant after adjusting for the multiple tests (Table S2).

## Discussion

Although MNSAID-UA is the most frequent clinical entity in HRs to drugs, it has received little attention so far [5], [6]. To date, the study of the genetic basis of NSAIDs hypersensitivity has focused mainly in AERD and CU, and followed the candidate gene approach considering genes related with the AA pathway (because of the biological plausibility) [47], [48]. Furthermore, the two GWAS in HRs published to date have been performed using a limited number of samples and only including patients with aspirin-induced asthma [33], [34]. One of such studies associated the non-synonymous polymorphism rs7572857 (Gly74Ser) in *CEP68* gene with changes in forced expiratory volume after aspirin administration, and proposed *CEP68* as a susceptibility gene for aspirin intolerance in asthmatics [33]. Here we evaluated the potential role of common genetic variants in this gene with MNSAID-UA susceptibility in a well-characterized group of patients. For this, we efficiently captured common variation of the gene by genotyping a set of 6 tagSNPs, including rs7572857, and boosted the study power by testing the association of 10 times more variants of this locus than in previous studies, by means of genotype imputation.

In the GWAS identifying *CEP68* as a key locus for HRs susceptibility associated with AERD [33], the association of rs7572857 was prominent and put forward as the potential causal variant affecting the polarity of the encoded protein and/or its function [33], [49]. *In silico* annotation showed that this polymorphism (Gly74Ser) is tolerable to human diseases, as the site is not highly conserved in mammals [33]. Here, we were able to find gene-level replication with MNSAID-UA susceptibility, although the effects observed for Spanish (A allele was protective) were opposite to those previously reported for Koreans (A allele was a risk) (Table 2) [33]. Because of this, the fact that many other variants of the region showed stronger association with MNSAID-UA than rs7572857 (Table 2), and the underlying LD in the gene region, it is difficult to judge if the associations observed are due to this SNP itself or to other nearby variants showing differential patterns of LD with it in the two populations. There is also the possibility that different functional variants exist in different populations, or that functional variants depend on other genetic or environmental factors (also differing between populations). The reason behind this observation is currently unknown, but it is clearly not specific for this complex trait or gene. Aiming to quantify how broadly the genetic associations described for a particular disease or trait will generalize to populations of different ancestries, a recent study by Carlson *et al.* [50] has explored a set of SNPs firmly associated with related complex traits in a large and diverse sample. Their observations suggest that the main factor contributing to such observation is the differential LD across continental populations between the associated SNPs of a study and the truly causal one(s) (or the synthetic alleles underlying the hit), which jeopardizes the generalization of association findings at SNP-level across populations, and can be particularly problematic for comparisons between Europeans and Asians [50].

It is important to note that there is one gene annotated in the 3′ flanking region (i.e. *RAB1A*) with variants in strong LD with *CEP68* variants. Note that the most consistent associations observed across MNSAID-UA, airway exacerbations and blended reactions localize in the vicinity of that region (e.g. rs61758846) (Table S2 and Figure 1). Therefore, to explore the possibility that *RAB1A* SNPs account for the association detected for *CEP68*, an *ad hoc* analysis with imputed data of the *RAB1A* gene allowed the identification of a total of 21 other common variants of the gene that were associated with MNSAID-UA at the same level of significance as that declared for *CEP68* (Table S5). However, this exploration did not reveal any other SNP with stronger significance in *RAB1A* than the top hit at *CEP68* (i.e. at the rs1050675). In addition, once the effects of the rs1050675 at *CEP68* were statistically accounted for using conditional regression analyses, none of the *RAB1A* SNPs remained significantly associated (Table S6). Further analyses performed considering all patients together, irrespective of the clinical group (data not shown), found that the most strongly associated SNP corresponded to rs61758846 at *CEP68* (p = 3.78×10^−7^), very close to those observed for rs1229 and rs1050675 (p = 4.07×10^−7^ and p = 4.40×10^−7^, respectively). Nevertheless, the functions of CEP68 protein are not fully understood, with the exception of its role in centrosome cohesion [51], [52], and in the epidermal growth factor (EGF) signaling pathway [53]. The latter may be involved in airway remodeling during allergic responses, by triggering the release of EGF ligands [54] or through the activation of its receptors by LTs [55]. As deduced from protein-protein network analysis, another potential relationship of CEP68 with hypersensitivity could be related to its putative associations with solute carrier family 1 member 4 (SLC1A4, which is inhibited by the potent bronchoconstrictor LTD4 [56]) and filamin A interacting protein 1 (FILIP1, recently associated with AERD susceptibility [57]) (Figure S1).

In summary, in this study we describe the association of *CEP68* variants and MNSAID-AU, showing that other variants different from those involved in the metabolic pathway of AA or in the homeostasis of mediators can be helpful for characterizing this pathology. Functional studies of the non-synonymous SNP rs7572857 are warranted to provide important insights into the genetic mechanisms underlying HRs to NSAIDs. Further replication studies in other populations and larger sample sizes are needed to confirm this association.

## Supporting Information

Figure S1 **Functional protein association network analysis for CEP68.** Interactions between CEP68 and other proteins were analysed using the STRING database (http://string-db.org/).
(DOC)

Table S1 **Quality control measures for genotyped tagging single nucleotide polymorphisms (tSNPs) in** ***CEP68*** **gene.**
(DOC)

Table S2 **Association of** ***CEP68*** **variants and hypersensitivity to MNSAIDs.** The tagSNPs and significant *p*-values after Bonferroni correction are shown in boldface. ORs values refer to the minor allele.
(DOC)

Table S3 **Association results for the 53 SNPs on** ***CEP68*** **for the three group comparisons and their corresponding q-values obtained assessing a false discovery rate (FDR).**
(DOC)

Table S4 **Results from the regression analyses of significantly associated CEP68 variants conditioning on the key SNP (rs1050675) testing for MNSAID-UA.**
(DOC)

Table S5 **Association of** ***RAB1A*** **imputed variants and hypersensitivity to NSAIDs.** Significant *p*-values after Bonferroni correction are shown in boldface. ORs values refer to the minor allele.
(DOC)

Table S6 **Results from the regression analyses on top associations at** ***CEP68*** **and** ***RAB1A*** **for MNSAID-AU conditioning on the key SNP from** ***CEP68*** **gene (rs1050675).**
(DOC)
