# Supplementary material for: Variants of CEP68 Gene Are Associated with Acute Urticaria/Angioedema Induced by Multiple Non-Steroidal Anti-Inflammatory Drugs
Source: PLoS One. 2014 Mar 11;9(3):e90966. doi: 10.1371/journal.pone.0090966 (PMC3949706; doi:10.1371/journal.pone.0090966)

**FIGURE S1.** **Functional protein association network analysis for CEP68**. Interactions between CEP68 and other proteins were analysed using the STRING database (<http://string-db.org/>).


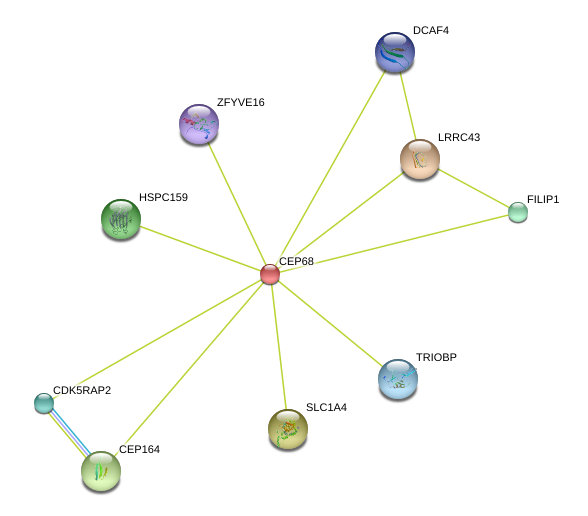

Supplement: Figure S1 — Functional protein association network analysis for CEP68. Interactions between CEP68 and other proteins were analysed using the STRING database (http://string-db.org/). (DOC) [file pone.0090966.s001.doc]
