# Supplementary material for: Variants of CEP68 Gene Are Associated with Acute Urticaria/Angioedema Induced by Multiple Non-Steroidal Anti-Inflammatory Drugs
Source: PLoS One. 2014 Mar 11;9(3):e90966. doi: 10.1371/journal.pone.0090966 (PMC3949706; doi:10.1371/journal.pone.0090966)
Supplement: Table S1 — Quality control measures for genotyped tagging single nucleotide polymorphisms (tSNPs) in CEP68 gene. (DOC) [file pone.0090966.s002.doc]

**Table S1.Quality control measures for genotyped tagging single nucleotide polymorphisms (tSNPs) in** *CEP68* **gene.**

| **tagSNPs** | **Change (Allele1, Allele 2)** | **Location (effect)** | **Position*** | **Call rate (%)** | **Genotype counts patients (n)** | | | **Genotype counts controls (n)** | | | **HWE patients** | **HWE controls** | **MAF patients** | **MAF controls** |
| --- | --- | --- | --- | --- | --- | --- | --- | --- | --- | --- | --- | --- | --- | --- |
|  |  |  |  |  | **1/1** | **1/2** | **2/2** | **1/1** | **1/2** | **2/2** |  |  |  |  |
| rs2249105 | A>G | Intron | 65287896 | 99.5 | 242 | 297 | 93 | 121 | 173 | 67 | 0.932 | 0.747 | 0.382 | 0.425 |
| rs2241161 | C>A | Intron | 65290842 | 99.6 | 498 | 131 | 4 | 270 | 85 | 6 | 0.217 | 1.000 | 0.110 | 0.134 |
| rs7572857 | G>A | Exon 2 (Gly74Ser) | 65296798 | 99.5 | 496 | 129 | 10 | 268 | 90 | 5 | 0.448 | 0.288 | 0.156 | 0.223 |
| rs10496123 | A>G | Intron 5 | 65309296 | 99.6 | 54 | 243 | 333 | 22 | 137 | 202 | 0.375 | 1.000 | 0.280 | 0.251 |
| rs1228 | T>C | 3´UTR | 65311555 | 100 | 288 | 280 | 67 | 145 | 167 | 51 | 1.000 | 0.882 | 0.326 | 0.371 |

**Abbreviations: HWE**, Hardy-Weinberg equilibrium p-values; **MAF**, minor allele frequency; **tagSNPs**, tagging single nucleotide polymorphisms**;** **3´UTR**, 3´ untranslated region.

* According to NCBI build 37.
