# Supplementary material for: Variants of CEP68 Gene Are Associated with Acute Urticaria/Angioedema Induced by Multiple Non-Steroidal Anti-Inflammatory Drugs
Source: PLoS One. 2014 Mar 11;9(3):e90966. doi: 10.1371/journal.pone.0090966 (PMC3949706; doi:10.1371/journal.pone.0090966)
Supplement: Table S2 — Association of CEP68 variants and hypersensitivity to MNSAIDs. The tagSNPs and significant p-values after Bonferroni correction are shown in boldface. ORs values refer to the minor allele. (DOC) [file pone.0090966.s003.doc]

**Table S2. Association of *CEP68* variants and hypersensitivity to MNSAIDs.** The tagSNPs and significant *p*-values after Bonferroni correction are shown in boldface. ORs values refer to the minor allele.

| **SNPs** | **Alleles (M, m)** | **MAF** | **Rsq** | **OR (95% CI)** | **P** | **OR (95% CI)** | **P** | **OR (95% CI)** | **P** |
| --- | --- | --- | --- | --- | --- | --- | --- | --- | --- |
|  |  |  |  | MNSAID-UA *vs* controls | | Airway exacerbations *vs* controls | | Blended pattern *vs* controls | |
| rs6728523 | G,C | 0.27 | 0.59 | 0.59 (0.43-0.80) | **6.15E-04** | 0.66 (0.42-1.03) | 6.57E-02 | 0.87 (0.58-1.32) | 5.20E-01 |
| rs2302647 | G,A | 0.27 | 0.59 | 0.59 (0.43-0.80) | **6.17E-04** | 0.66 (0.42-1.03) | 6.58E-02 | 0.87 (0.58-1.32) | 5.20E-01 |
| rs74181299 | T,C | 0.42 | 0.87 | 0.81 (0.65-1.01) | 5.61E-02 | 0.84 (0.61-1.16) | 2.88E-01 | 0.91 (0.67-1.25) | 5.72E-01 |
| rs2540949 | A,T | 0.41 | 0.90 | 0.81 (0.65-1.00) | 5.35E-02 | 0.85 (0.61-1.16) | 3.03E-01 | 0.92 (0.68-1.24) | 5.79E-01 |
| rs2540948 | T,C | 0.38 | 0.78 | 0.84 (0.67-1.07) | 1.57E-01 | 0.86 (0.60-1.23) | 4.10E-01 | 0.94 (0.67-1.32) | 7.27E-01 |
| rs2540947 | A,G | 0.14 | 0.33 | 1.37 (0.83-2.25) | 2.15E-01 | 1.35 (0.64-2.83) | 4.32E-01 | 0.93 (0.44-2.00) | 8.58E-01 |
| rs62140397 | C,T | 0.14 | 0.33 | 1.37 (0.83-2.25) | 2.15E-01 | 1.35 (0.64-2.83) | 4.32E-01 | 0.93 (0.44-1.99) | 8.57E-01 |
| rs2723080 | C,T | 0.14 | 0.33 | 1.37 (0.83-2.27) | 2.15E-01 | 1.35 (0.64-2.86) | 4.34E-01 | 0.93 (0.43-2.01) | 8.58E-01 |
| rs2723081 | G,C | 0.14 | 0.33 | 1.37 (0.83-2.25) | 2.16E-01 | 1.34 (0.64-2.82) | 4.33E-01 | 0.93 (0.44-1.99) | 8.58E-01 |
| rs2540946 | C,G | 0.14 | 0.34 | 1.34 (0.83-2.18) | 2.31E-01 | 1.32 (0.64-2.72) | 4.46E-01 | 0.93 (0.45-1.94) | 8.47E-01 |
| **rs2249105** | A,G | 0.40 | - | 0.78 (0.63-0.96) | 1.75E-02 | 0.89 (0.65-1.20) | 4.38E-01 | 1.00 (0.75-1.33) | 5.90E-01 |
| rs2723082 | C,T | 0.32 | 0.70 | 0.81 (0.63-1.06) | 1.30E-01 | 0.91 (0.61-1.35) | 6.26E-01 | 0.93 (0.63-1.37) | 7.10E-01 |
| rs2540945 | A,G | 0.35 | 0.75 | 0.75 (0.59-0.97) | 2.86E-02 | 0.87 (0.60-1.26) | 4.60E-01 | 0.89 (0.62-1.28) | 5.33E-01 |
| rs2723083 | C,T | 0.32 | 0.69 | 0.81 (0.62-1.06) | 1.30E-01 | 0.92 (0.61-1.37) | 6.70E-01 | 0.93 (0.63-1.37) | 7.07E-01 |
| **rs2241161** | C,A | 0.38 | - | 0.75 (0.54-1.03) | 1.70E-02 | 0.83 (0.52-1.33) | 4.44E-01 | 0.87 (0.56-1.35) | 5.30E-01 |
| rs2241160 | A,G | 0.38 | 0.64 | 0.73 (0.56-0.96) | 2.59E-02 | 0.86 (0.58-1.28) | 4.65E-01 | 0.89 (0.61-1.30) | 5.39E-01 |
| rs2901749 | G,T | 0.19 | 0.57 | 0.51 (0.35-0.74) | **3.74E-04** | 0.65 (0.38-1.12) | 1.18E-01 | 0.81 (0.50-1.32) | 4.01E-01 |
| rs2080385 | G,T | 0.22 | 0.54 | 0.43 (0.30-0.62) | **6.61E-06** | 0.60 (0.35-1.01) | 5.45E-02 | 0.75 (0.46-1.21) | 2.39E-01 |
| rs75678687 | G,A | 0.19 | 0.58 | 0.51 (0.35-0.73) | **2.59E-04** | 0.64 (0.38-1.10) | 1.06E-01 | 0.79 (0.49-1.29) | 3.54E-01 |
| rs62140398 | C,T | 0.12 | 0.39 | 1.60 (0.97-2.64) | 6.51E-02 | 1.78 (0.87-3.65) | 1.18E-01 | 1.12 (0.53-2.38) | 7.68E-01 |
| rs79157909 | T,G | 0.16 | 0.42 | 0.46 (0.29-0.72) | **8.30E-04** | 0.62 (0.32-1.20) | 1.53E-01 | 0.81 (0.44-1.48) | 4.95E-01 |
| rs2252867 | T,C | 0.34 | 0.69 | 0.69 (0.53-0.91) | 7.16E-03 | 0.84 (0.57-1.25) | 4.00E-01 | 0.82 (0.56-1.21) | 3.24E-01 |
| **rs7572857** | G,A | 0.14 | - | 0.55 (0.42-0.73) | **1.67E-05** | 0.74 (0.50-1.10) | 1.35E-01 | 0.81 (0.56-1.17) | 2.61E-01 |
| rs2723084 | C,T | 0.12 | 0.39 | 1.60 (0.97-2.64) | 6.36E-02 | 1.78 (0.87-3.65) | 1.15E-01 | 1.13 (0.54-2.40) | 7.43E-01 |
| rs2723085 | C,G | 0.31 | 0.68 | 0.76 (0.57-1.00) | 4.71E-02 | 0.89 (0.59-1.33) | 5.66E-01 | 0.86 (0.58-1.27) | 4.44E-01 |
| rs2723086 | C,T | 0.31 | 0.68 | 0.76 (0.57-1.00) | 4.69E-02 | 0.89 (0.59-1.33) | 5.64E-01 | 0.86 (0.58-1.27) | 4.42E-01 |
| rs2723087 | T,A | 0.31 | 0.68 | 0.76 (0.57-1.00) | 4.62E-02 | 0.89 (0.59-1.33) | 5.62E-01 | 0.86 (0.58-1.27) | 4.38E-01 |
| rs17849707 | A,G | 0.20 | 0.57 | 0.49 (0.34-0.70) | **1.09E-04** | 0.62 (0.37-1.06) | 8.08E-02 | 0.75 (0.46-1.22) | 2.53E-01 |
| rs2723088 | C,A | 0.12 | 0.39 | 1.60 (0.97-2.64) | 6.43E-02 | 1.78 (0.87-3.65) | 1.16E-01 | 1.14 (0.54-2.41) | 7.35E-01 |
| rs2723089 | G,C | 0.12 | 0.39 | 1.60 (0.97-2.64) | 6.44E-02 | 1.78 (0.87-3.65) | 1.16E-01 | 1.14 (0.54-2.41) | 7.34E-01 |
| rs12621608 | C,T | 0.16 | 0.49 | 0.43 (0.29-0.65) | **5.85E-05** | 0.57 (0.32-1.03) | 6.42E-02 | 0.57 (0.32-1.00) | 5.18E-02 |
| rs2723090 | G,A | 0.12 | 0.39 | 1.60 (0.97-2.63) | 6.62E-02 | 1.78 (0.87-3.64) | 1.17E-01 | 1.14 (0.54-2.42) | 7.23E-01 |
| rs2723091 | C,T | 0.29 | 0.66 | 0.76 (0.58-1.01) | 5.57E-02 | 0.89 (0.59-1.34) | 5.65E-01 | 0.76 (0.50-1.14) | 1.82E-01 |
| rs6546123 | C,T | 0.32 | 0.64 | 0.68 (0.52-0.90) | 7.03E-03 | 0.83 (0.55-1.25) | 3.79E-01 | 0.72 (0.48-1.08) | 1.10E-01 |
| rs6732556 | A,G | 0.12 | 0.39 | 1.56 (0.95-2.58) | 7.97E-02 | 1.74 (0.85-3.59) | 1.32E-01 | 1.15 (0.54-2.44) | 7.15E-01 |
| rs76221156 | C,T | 0.11 | 0.61 | 0.34 (0.22-0.53) | **1.74E-06** | 0.46 (0.24-0.87) | 1.81E-02 | 0.51 (0.28-0.93) | 2.80E-02 |
| rs6736728 | T,C | 0.29 | 0.68 | 0.76 (0.58-1.00) | 5.30E-02 | 0.88 (0.59-1.33) | 5.47E-01 | 0.75 (0.50-1.13) | 1.73E-01 |
| rs6546124 | C,T | 0.29 | 0.68 | 0.76 (0.58-1.00) | 5.27E-02 | 0.88 (0.59-1.32) | 5.44E-01 | 0.75 (0.50-1.13) | 1.71E-01 |
| rs6741255 | T,C | 0.29 | 0.69 | 0.76 (0.58-1.00) | 5.23E-02 | 0.88 (0.59-1.32) | 5.40E-01 | 0.76 (0.51-1.13) | 1.70E-01 |
| rs1894874 | T,C | 0.12 | 0.59 | 0.34 (0.22-0.52) | **1.34E-06** | 0.45 (0.23-0.86) | 1.60E-02 | 0.49 (0.27-0.90) | 2.52E-02 |
| rs1541576 | C,T | 0.12 | 0.39 | 1.53 (0.94-2.50) | 8.88E-02 | 1.71 (0.84-3.47) | 1.40E-01 | 1.16 (0.56-2.41) | 6.98E-01 |
| rs113359765 | C,T | 0.11 | 0.59 | 0.34 (0.22-0.52) | **1.20E-06** | 0.44 (0.23-0.86) | 1.52E-02 | 0.48 (0.26-0.89) | 2.01E-02 |
| rs6546125 | A,C | 0.17 | 0.48 | 0.44 (0.29-0.65) | **4.54E-05** | 0.57 (0.32-1.02) | 5.75E-02 | 0.55 (0.31-0.96) | 3.48E-02 |
| **rs10496123** | A,G | 0.26 | - | 0.86 (0.87-1.41) | 2.04E-01 | 0.76 (0.95-1.85) | 9.61E-02 | 0.98 (0.84-1.64) | 9.17E-01 |
| rs78945874 | A,G | 0.11 | 0.58 | 0.33 (0.22-0.52) | **1.17E-06** | 0.44 (0.23-0.85) | 1.46E-02 | 0.48 (0.26-0.89) | 1.90E-02 |
| rs1420183 | C,T | 0.29 | 0.71 | 0.77 (0.59-1.01) | 5.56E-02 | 0.87 (0.59-1.29) | 4.91E-01 | 0.75 (0.51-1.11) | 1.52E-01 |
| **rs3732098** | T,C | 0.12 | - | 1.10 (0.19-6.31) | 9.17E-01 | 1.32 (0.09-19.10) | 8.37E-01 | 1.07 (0.08-13.86) | 9.60E-01 |
| rs1050676 | T,A | 0.30 | 0.72 | 0.78 (0.60-1.01) | 6.00E-02 | 0.86 (0.59-1.28) | 4.64E-01 | 0.75 (0.51-1.10) | 1.44E-01 |
| rs1050675 | A,G | 0.11 | 0.56 | 0.33 (0.21-0.52) | **1.13E-06** | 0.43 (0.22-0.83) | 1.19E-02 | 0.46 (0.25-0.86) | 1.48E-02 |
| rs1229 | G,A | 0.11 | 0.56 | 0.33 (0.21-0.52) | **1.14E-06** | 0.42 (0.22-0.82) | 1.13E-02 | 0.46 (0.25-0.85) | 1.37E-02 |
| **rs1228** | T,C | 0.30 | - | 0.78 (0.63-0.96) | 2.14E-02 | 0.87 (0.64-1.20) | 3.95E-01 | 0.90 (0.67-1.22) | 5.09E-01 |
| rs4671638 | T,C | 0.24 | 0.57 | 0.66 (0.48-0.90) | 8.93E-03 | 0.74 (0.47-1.18) | 2.11E-01 | 0.65 (0.41-1.03) | 6.75E-02 |
| rs61758846 | G,C | 0.12 | 0.52 | 0.32 (0.21-0.51) | **1.16E-06** | 0.41 (0.21-0.81) | 1.06E-02 | 0.44 (0.23-0.84) | 1.28E-02 |

**Abbreviations: CI**, confidence interval; **MAF**, minor allele frequency; **M, m**, major and minor alleles, respectively; NSAIDs, non-steroidal anti-inflammatory drugs; **Rsq**, squared correlation between imputed and true genotypes; **OR**, odds ratio.
