# Supplementary material for: Variants of CEP68 Gene Are Associated with Acute Urticaria/Angioedema Induced by Multiple Non-Steroidal Anti-Inflammatory Drugs
Source: PLoS One. 2014 Mar 11;9(3):e90966. doi: 10.1371/journal.pone.0090966 (PMC3949706; doi:10.1371/journal.pone.0090966)
Supplement: Table S3 — Association results for the 53 SNPs on CEP68 for the three group comparisons and their corresponding q-values obtained assessing a false discovery rate (FDR). (DOC) [file pone.0090966.s004.doc]

**Table S3. Association results for the 53 SNPs on *CEP68* for the three group comparisons and their corresponding q-values obtained assessing a false discovery rate (FDR).**

|  | **MNSAID-UA vs controls** | | **Airway ex. vs controls** | | **Blended pattern vs controls** | |
| --- | --- | --- | --- | --- | --- | --- |
| SNP | **p-value** | **q-value** | **p-value** | **q-value** | **p-value** | **q-value** |
| rs6728523 | **0.000615** | **0.006131** | 0.065650 | 0.169186 | 0.520000 | 0.656695 |
| rs2302647 | **0.000617** | **0.006131** | 0.065810 | 0.169186 | 0.520400 | 0.656695 |
| rs74181299 | 0.056140 | 0.169186 | 0.287900 | 0.462385 | 0.571700 | 0.663506 |
| rs2540949 | 0.053540 | 0.169186 | 0.302800 | 0.481452 | 0.578700 | 0.666763 |
| rs2540948 | 0.156600 | 0.292934 | 0.409800 | 0.597782 | 0.727200 | 0.784329 |
| rs2540947 | 0.214800 | 0.364854 | 0.432200 | 0.600562 | 0.858400 | 0.869335 |
| rs62140397 | 0.214800 | 0.364854 | 0.432100 | 0.600562 | 0.856700 | 0.869335 |
| rs2723080 | 0.215200 | 0.364854 | 0.433500 | 0.600562 | 0.857700 | 0.869335 |
| rs2723081 | 0.215700 | 0.364854 | 0.433300 | 0.600562 | 0.858400 | 0.869335 |
| rs2540946 | 0.231100 | 0.386788 | 0.445700 | 0.600562 | 0.847400 | 0.869335 |
| rs2249105 | 0.050820 | 0.169186 | 0.325800 | 0.507865 | 0.589700 | 0.674549 |
| rs2723082 | 0.129600 | 0.264665 | 0.626200 | 0.711184 | 0.710100 | 0.784069 |
| rs2540945 | 0.028630 | 0.126449 | 0.459500 | 0.611296 | 0.533200 | 0.661604 |
| rs2723083 | 0.130000 | 0.264665 | 0.669600 | 0.755081 | 0.706700 | 0.784069 |
| rs2241161 | 0.030120 | 0.129435 | 0.484500 | 0.631439 | 0.565900 | 0.661604 |
| rs2241160 | 0.025880 | 0.121027 | 0.465200 | 0.611296 | 0.539400 | 0.661604 |
| rs2901749 | **0.000374** | **0.004248** | 0.118300 | 0.247496 | 0.400600 | 0.589772 |
| rs2080385 | **0.000007** | **0.000139** | 0.054520 | 0.169186 | 0.238800 | 0.395513 |
| rs75678687 | **0.000259** | **0.003168** | 0.106300 | 0.244952 | 0.354400 | 0.547084 |
| rs62140398 | 0.065130 | 0.169186 | 0.117700 | 0.247496 | 0.767700 | 0.808373 |
| rs79157909 | **0.000830** | **0.007763** | 0.152600 | 0.288850 | 0.495100 | 0.634846 |
| rs2252867 | 0.007157 | 0.059893 | 0.399900 | 0.589772 | 0.324200 | 0.507865 |
| rs7572857 | **0.000016** | **0.000283** | 0.038510 | 0.157002 | 0.242800 | 0.397992 |
| rs2723084 | 0.063630 | 0.169186 | 0.115000 | 0.247496 | 0.742500 | 0.787050 |
| rs2723085 | 0.047090 | 0.169186 | 0.565700 | 0.661604 | 0.443600 | 0.600562 |
| rs2723086 | 0.046870 | 0.169186 | 0.564300 | 0.661604 | 0.442200 | 0.600562 |
| rs2723087 | 0.046230 | 0.169186 | 0.562100 | 0.661604 | 0.437600 | 0.600562 |
| rs17849707 | **0.000109** | **0.001444** | 0.080780 | 0.194606 | 0.252900 | 0.410317 |
| rs2723088 | 0.064270 | 0.169186 | 0.115800 | 0.247496 | 0.735000 | 0.784329 |
| rs2723089 | 0.064410 | 0.169186 | 0.115800 | 0.247496 | 0.734100 | 0.784329 |
| rs12621608 | **0.000058** | **0.000838** | 0.064180 | 0.169186 | 0.051800 | 0.169186 |
| rs2723090 | 0.066220 | 0.169186 | 0.116600 | 0.247496 | 0.723400 | 0.784329 |
| rs2723091 | 0.055700 | 0.169186 | 0.565300 | 0.661604 | 0.182300 | 0.325682 |
| rs6546123 | 0.007027 | 0.059893 | 0.378500 | 0.573157 | 0.109700 | 0.247496 |
| rs6732556 | 0.079690 | 0.194606 | 0.131500 | 0.264665 | 0.715100 | 0.784144 |
| rs76221156 | **0.000002** | **0.000045** | 0.018140 | 0.096142 | 0.027950 | 0.126449 |
| rs6736728 | 0.053040 | 0.169186 | 0.547400 | 0.661604 | 0.172800 | 0.312218 |
| rs6546124 | 0.052710 | 0.169186 | 0.544000 | 0.661604 | 0.171400 | 0.312218 |
| rs6741255 | 0.052290 | 0.169186 | 0.539700 | 0.661604 | 0.169700 | 0.312218 |
| rs1894874 | **0.000001** | **0.000027** | 0.016010 | 0.087779 | 0.022460 | 0.108216 |
| rs1541576 | 0.088830 | 0.210806 | 0.139500 | 0.273833 | 0.698000 | 0.781563 |
| rs113359765 | **0.000001** | **0.000027** | 0.015240 | 0.086541 | 0.020060 | 0.099673 |
| rs6546125 | **0.000045** | **0.000716** | 0.057470 | 0.169186 | 0.034810 | 0.145652 |
| rs10496123 | 0.392900 | 0.589350 | 0.101300 | 0.236863 | 0.362600 | 0.554360 |
| rs78945874 | **0.000001** | **0.000027** | 0.014610 | 0.086541 | 0.019020 | 0.097554 |
| rs1420183 | 0.055580 | 0.169186 | 0.491200 | 0.634846 | 0.152400 | 0.288850 |
| rs1050676 | 0.059950 | 0.169186 | 0.464000 | 0.611296 | 0.143900 | 0.279026 |
| rs1050675 | **0.000001** | **0.000027** | 0.011920 | 0.082403 | 0.014760 | 0.086541 |
| rs1229 | **0.000001** | **0.000027** | 0.011300 | 0.081668 | 0.013700 | 0.086541 |
| rs1228 | 0.068100 | 0.169186 | 0.434300 | 0.600562 | 0.136600 | 0.271493 |
| rs4671638 | 0.008929 | 0.070986 | 0.210500 | 0.364854 | 0.067480 | 0.169186 |
| rs61758846 | **0.000001** | **0.000027** | 0.010550 | 0.079879 | 0.012840 | 0.085065 |
| rs3732098 | 0.917500 | 0.923307 | 0.836900 | 0.869335 | 0.960200 | 0.960200 |
| In bold, the significant associations according to a Bonferroni correction (p-values) and to a 5% FDR (q-value). | | | | | | |
