# Supplementary material for: Variants of CEP68 Gene Are Associated with Acute Urticaria/Angioedema Induced by Multiple Non-Steroidal Anti-Inflammatory Drugs
Source: PLoS One. 2014 Mar 11;9(3):e90966. doi: 10.1371/journal.pone.0090966 (PMC3949706; doi:10.1371/journal.pone.0090966)
Supplement: Table S4 — Results from the regression analyses of significantly associated CEP68 variants conditioning on the key SNP (rs1050675) testing for MNSAID-UA. (DOC) [file pone.0090966.s005.doc]

**Table S4. Results from the regression analyses of significantly associated *CEP68*** variants conditioning on the key SNP (rs1050675) testing for MNSAID-UA.

| **SNP** | **p-value** |
| --- | --- |
| rs6728523 | 0.140 |
| rs2302647 | 0.141 |
| rs2901749 | 0.244 |
| rs2080385 | 0.257 |
| rs75678687 | 0.276 |
| rs79157909 | 0.312 |
| rs7572857 | 0.921 |
| rs17849707 | 0.352 |
| rs12621608 | 0.111 |
| rs76221156 | 0.707 |
| rs1894874 | 0.882 |
| rs113359765 | 0.967 |
| rs6546125 | 0.143 |
| rs78945874 | 0.949 |
| rs1229 | 0.906 |
| rs61758846 | 0.905 |
