# Supplementary material for: Variants of CEP68 Gene Are Associated with Acute Urticaria/Angioedema Induced by Multiple Non-Steroidal Anti-Inflammatory Drugs
Source: PLoS One. 2014 Mar 11;9(3):e90966. doi: 10.1371/journal.pone.0090966 (PMC3949706; doi:10.1371/journal.pone.0090966)
Supplement: Table S5 — Association of RAB1A imputed variants and hypersensitivity to NSAIDs. Significant p-values after Bonferroni correction are shown in boldface. ORs values refer to the minor allele. (DOC) [file pone.0090966.s006.doc]

**Table S5. Association of *RAB1A* imputed variants and hypersensitivity to NSAIDs.** Significant *p*-values after Bonferroni correction are shown in boldface. ORs values refer to the minor allele.

| **SNPs** | **Alleles (M. m)** | **MAF** | **Rsq** | **OR (95% CI)** | **P** | **OR (95% CI)** | **P** | **OR (95% CI)** | **P** |
| --- | --- | --- | --- | --- | --- | --- | --- | --- | --- |
|  |  |  |  | MNSAID-UA *vs* controls | | Airway exacerbations *vs* controls | | Blended pattern *vs* controls | |
| rs1420184 | C.G | 0.27 | 0.69 | 0.76 (0.48-1.04) | 5.52E-02 | 0.90 (0.69-1.31) | 6.20E-01 | 0.76 (0.35-1.17) | 1.93E-01 |
| rs1420185 | A.G | 0.30 | 0.66 | 0.69 (0.41-0.97) | 8.47E-03 | 0.85 (0.64-1.26) | 4.33E-01 | 0.72 (0.32-1.13) | 1.23E-01 |
| rs1055903 | A.G | 0.27 | 0.69 | 0.76 (0.48-1.04) | 5.54E-02 | 0.90 (0.69-1.31) | 6.21E-01 | 0.76 (0.35-1.17) | 1.93E-01 |
| rs112029776 | A.G | 0.11 | 0.59 | 0.35 (0.10-3.33) | **3.05E-06** | 0.45 (0.11-1.12) | 2.04E-02 | 0.48 (0.14-1.11) | 2.28E-02 |
| rs7592816 | G.A | 0.11 | 0.38 | 1.54 (1.01-2.06) | 1.06E-01 | 1.83 (1.45-2.58) | 1.14E-01 | 1.22 (0.45-1.99) | 6.11E-01 |
| rs113413623 | G.A | 0.11 | 0.59 | 0.35 (0.10-3.34) | **3.09E-06** | 0.45 (0.11-1.12) | 2.05E-02 | 0.48 (0.14-1.11) | 2.29E-02 |
| rs113506850 | T.G | 0.10 | 0.58 | 0.34 (0.12-3.44) | **3.10E-06** | 0.45 (0.10-1.13) | 2.10E-02 | 0.48 (0.17-1.12) | 2.31E-02 |
| rs7607025 | G.A | 0.22 | 0.54 | 0.62 (0.29-0.96) | 5.70E-03 | 0.78 (0.53-1.27) | 3.11E-01 | 0.67 (0.18-1.15) | 9.95E-02 |
| rs7607039 | G.A | 0.22 | 0.54 | 0.62 (0.29-0.96) | 5.70E-03 | 0.78 (0.53-1.27) | 3.10E-01 | 0.67 (0.18-1.15) | 9.96E-02 |
| rs6713746 | C.G | 0.27 | 0.68 | 0.76 (0.48-1.04) | 5.51E-02 | 0.90 (0.69-1.31) | 6.20E-01 | 0.76 (0.35-1.17) | 1.92E-01 |
| rs6729273 | C.T | 0.11 | 0.38 | 1.54 (1.02-2.06) | 1.06E-01 | 1.83 (1.45-2.59) | 1.14E-01 | 1.22 (0.45-1.99) | 6.13E-01 |
| rs76210337 | A.G | 0.11 | 0.59 | 0.34 (0.10-3.35) | **3.06E-06** | 0.45 (0.11-1.12) | 2.04E-02 | 0.48 (0.14-1.11) | 2.28E-02 |
| rs75263790 | G.T | 0.11 | 0.59 | 0.34 (0.10-3.35) | **3.04E-06** | 0.45 (0.11-1.12) | 2.03E-02 | 0.48 (0.14-1.11) | 2.28E-02 |
| rs9784118 | C.T | 0.16 | 0.58 | 1.35 (0.99-1.72) | 1.02E-01 | 1.53 (1.26-2.05) | 1.10E-01 | 1.15 (0.62-1.69) | 6.06E-01 |
| rs9784068 | C.A | 0.11 | 0.58 | 0.34 (0.10-3.35) | **3.09E-06** | 0.45 (0.11-1.12) | 2.05E-02 | 0.48 (0.14-1.11) | 2.29E-02 |
| rs4671115 | T.C | 0.16 | 0.57 | 1.35 (0.99-1.72) | 1.01E-01 | 1.53 (1.27-2.05) | 1.10E-01 | 1.15 (0.61-1.69) | 6.06E-01 |
| rs17040050 | A.G | 0.27 | 0.68 | 0.76 (0.48-1.04) | 5.52E-02 | 0.90 (0.69-1.31) | 6.20E-01 | 0.76 (0.35-1.179 | 1.93E-01 |
| rs6725335 | A.C | 0.16 | 0.57 | 1.35 (0.99-1.72) | 1.01E-01 | 1.53 (1.26-2.05) | 1.10E-01 | 1.15 (0.61-1.69) | 6.06E-01 |
| rs12472718 | A.G | 0.30 | 0.65 | 0.69 (0.41-0.97) | 8.47E-03 | 0.85 (0.64-1.26) | 4.33E-01 | 0.72 (0.31-1.13) | 1.23E-01 |
| rs113467506 | C.G | 0.11 | 0.58 | 0.34 (0.11-3.36) | **3.06E-06** | 0.45 (0.11-1.12) | 2.05E-02 | 0.48 (0.14-1.11) | 2.28E-02 |
| rs7579489 | C.G | 0.27 | 0.67 | 0.76 (0.48-1.04) | 5.55E-02 | 0.90 (0.69-1.31) | 6.16E-01 | 0.76 (0.35-1.17) | 1.92E-01 |
| rs10195328 | A.G | 0.16 | 0.57 | 1.36 (0.99-1.72) | 1.01E-01 | 1.53 (1.27-2.06) | 1.10E-01 | 1.15 (0.61-1.69) | 6.06E-01 |
| rs12713529 | C.G | 0.16 | 0.57 | 1.36 (0.99-1.72) | 1.01E-01 | 1.53 (1.27-2.06) | 1.10E-01 | 1.15 (0.61-1.69) | 6.07E-01 |
| rs75479685 | A.G | 0.11 | 0.58 | 0.34 (0.11-3.36) | **3.06E-06** | 0.45 (0.11-1.12) | 2.04E-02 | 0.48 (0.15-1.11) | 2.28E-02 |
| rs7563866 | G.A | 0.28 | 0.65 | 0.76 (0.48-1.04) | 5.66E-02 | 0.90 (0.69-1.32) | 6.24E-01 | 0.76 (0.34-1.17) | 1.92E-01 |
| rs7590690 | A.G | 0.27 | 0.67 | 0.76 (0.48-1.04) | 5.55E-02 | 0.90 (0.69-1.31) | 6.21E-01 | 0.76 (0.35-1.17) | 1.93E-01 |
| rs111245132 | T.G | 0.11 | 0.58 | 0.34 (0.11-3.36) | **3.09E-06** | 0.45 (0.11-1.12) | 2.05E-02 | 0.48 (0.15-1.11) | 2.28E-02 |
| rs6741843 | T.C | 0.16 | 0.57 | 1.36 (0.99-1.72) | 1.01E-01 | 1.53 (1.27-2.06) | 1.11E-01 | 1.15 (0.61-1.69) | 6.06E-01 |
| rs2302631 | G.T | 0.27 | 0.66 | 0.76 (0.48-1.04) | 5.56E-02 | 0.90 (0.69-1.32) | 6.32E-01 | 0.76 (0.35-1.17) | 1.94E-01 |
| rs10187798 | G.A | 0.17 | 0.56 | 1.36 (1.00-1.73) | 9.67E-02 | 1.55 (1.28-2.08) | 1.03E-01 | 1.16 (0.62-1.70) | 5.94E-01 |
| rs13005414 | A.G | 0.27 | 0.66 | 0.76 (0.48-1.04) | 5.60E-02 | 0.90 (0.69-1.32) | 6.31E-01 | 0.76 (0.34-1.18) | 1.94E-01 |
| rs10165691 | G.T | 0.17 | 0.56 | 1.36 (1.00-1.73) | 9.65E-02 | 1.55 (1.28-2.08) | 1.03E-01 | 1.16 (0.62-1.70) | 5.94E-01 |
| rs6736982 | T.C | 0.27 | 0.66 | 0.76 (0.48-1.04) | 5.59E-02 | 0.90 (0.69-1.32) | 6.33E-01 | 0.76 (0.34-1.18) | 1.94E-01 |
| rs13421845 | A.G | 0.30 | 0.63 | 0.68 (0.40-0.97) | 8.41E-03 | 0.85 (0.64-1.27) | 4.41E-01 | 0.72 (0.31-1.14) | 1.24E-01 |
| rs11885480 | C.T | 0.17 | 0.55 | 1.36 (1.00-1.73) | 9.69E-02 | 1.55 (1.28-2.08) | 1.03E-01 | 1.16 (0.62-1.70) | 5.95E-01 |
| rs9797989 | C.T | 0.27 | 0.66 | 0.76 (0.48-1.04) | 5.56E-02 | 0.90 (0.69-1.32) | 6.31E-01 | 0.76 (0.34-1.18) | 1.94E-01 |
| rs111778391 | G.A | 0.11 | 0.57 | 0.34 (0.11-3.38) | **3.10E-06** | 0.45 (0.10-1.13) | 2.05E-02 | 0.48 (0.15-1.11) | 2.29E-02 |
| rs10207159 | A.G | 0.12 | 0.36 | 1.56 (1.03-2.09) | 9.94E-02 | 1.89 (1.50-2.65) | 1.04E-01 | 1.24 (0.45-2.02) | 5.95E-01 |
| rs4671116 | A.G | 0.27 | 0.66 | 0.76 (0.48-1.04) | 5.59E-02 | 0.90 (0.69-1.32) | 6.32E-01 | 0.76 (0.34-1.17) | 1.94E-01 |
| rs11126028 | C.G | 0.27 | 0.65 | 0.76 (0.48-1.04) | 5.58E-02 | 0.90 (0.69-1.32) | 6.31E-01 | 0.76 (0.34-1.17) | 1.94E-01 |
| rs11126029 | T.C | 0.27 | 0.65 | 0.76 (0.47-1.04) | 5.58E-02 | 0.90 (0.69-1.32) | 6.32E-01 | 0.76 (0.34-1.18) | 1.94E-01 |
| rs60136636 | A.G | 0.30 | 0.62 | 0.68 (0.40-0.97) | 8.42E-03 | 0.85 (0.63-1.27) | 4.41E-01 | 0.72 (0.30-1.14) | 1.24E-01 |
| rs34374525 | G.A | 0.17 | 0.54 | 1.34 (0.97-1.70) | 1.22E-01 | 1.53 (1.26-2.06) | 1.18E-01 | 1.14 (0.59-1.69) | 6.41E-01 |
| rs62140404 | A.C | 0.30 | 0.41 | 1.22 (0.89-1.15) | 2.32E-01 | 0.79 (0.53-1.30) | 3.64E-01 | 1.14 (0.65-1.63) | 6.09E-01 |
| rs10176281 | T.A | 0.11 | 0.57 | 0.34 (0.11-3.39) | **3.08E-06** | 0.45 (0.10-1.13) | 2.05E-02 | 0.48 (0.16-1.11) | 2.28E-02 |
| rs113683435 | G.A | 0.11 | 0.57 | 0.34 (0.11-3.39) | **3.10E-06** | 0.45 (0.10-1.13) | 2.04E-02 | 0.48 (0.16-1.11) | 2.28E-02 |
| rs12472234 | G.A | 0.17 | 0.55 | 1.37 (1.00-1.73) | 9.72E-02 | 1.55 (1.28-2.09) | 1.04E-01 | 1.16 (0.61-1.70) | 5.96E-01 |
| rs6756585 | A.G | 0.27 | 0.65 | 0.76 (0.47-1.04) | 5.55E-02 | 0.90 (0.69-1.32) | 6.31E-01 | 0.76 (0.34-1.18) | 1.94E-01 |
| rs56725299 | G.C | 0.11 | 0.57 | 0.34 (0.12-3.40) | **3.06E-06** | 0.45 (0.10-1.13) | 2.03E-02 | 0.48 (0.16-1.11) | 2.28E-02 |
| rs6757520 | T.C | 0.17 | 0.55 | 1.37 (1.00-1.73) | 9.69E-02 | 1.56 (1.28-2.09) | 1.03E-01 | 1.16 (0.61-1.70) | 5.94E-01 |
| rs17029863 | T.C | 0.11 | 0.57 | 0.34 (0.12-3.40) | **3.06E-06** | 0.45 (0.10-1.13) | 2.04E-02 | 0.48 (0.16-1.11) | 2.28E-02 |
| rs13006381 | A.G | 0.39 | 0.55 | 0.66 (0.37-0.95) | 5.44E-03 | 0.81 (0.60-1.23) | 3.33E-01 | 0.81 (0.40-1.22) | 3.13E-01 |
| rs1019303 | C.T | 0.17 | 0.55 | 1.37 (1.00-1.74) | 9.67E-02 | 1.56 (1.29-2.09) | 1.03E-01 | 1.16 (0.61-1.71) | 5.94E-01 |
| rs1019304 | G.A | 0.12 | 0.35 | 1.57 (1.03-2.10) | 9.92E-02 | 1.89 (1.50-2.66) | 1.04E-01 | 1.24 (0.45-2.03) | 5.96E-01 |
| rs1019305 | C.T | 0.17 | 0.54 | 1.37 (1.00-1.74) | 9.69E-02 | 1.56 (1.29-2.09) | 1.03E-01 | 1.16 (0.61-1.71) | 5.95E-01 |
| rs13409078 | T.C | 0.11 | 0.56 | 0.34 (0.12-3.42) | **3.06E-06** | 0.45 (0.10-1.13) | 2.03E-02 | 0.48 (0.16-1.11) | 2.28E-02 |
| rs1558658 | A.G | 0.11 | 0.56 | 0.34 (0.12-3.42) | **3.07E-06** | 0.45 (0.10-1.13) | 2.03E-02 | 0.48 (0.16-1.11) | 2.28E-02 |
| rs1558659 | C.T | 0.17 | 0.54 | 1.37 (1.00-1.74) | 9.70E-02 | 1.56 (1.29-2.09) | 1.03E-01 | 1.16 (0.61-1.71) | 5.94E-01 |
| rs1558660 | T.C | 0.17 | 0.54 | 1.37 (1.00-1.74) | 9.71E-02 | 1.56 (1.29-2.09) | 1.03E-01 | 1.16 (0.61-1.71) | 5.94E-01 |
| rs28392943 | T.C | 0.89 | 0.56 | 0.34 (0.12-3.42) | **3.08E-06** | 0.45 (0.10-1.13) | 2.04E-02 | 0.48 (0.16-1.12) | 2.28E-02 |
| rs13383649 | G.C | 0.89 | 0.56 | 0.34 (0.12-3.42) | **3.07E-06** | 0.45 (0.10-1.13) | 2.04E-02 | 0.48 (0.16-1.12) | 2.28E-02 |
| rs6546127 | G.T | 0.11 | 0.36 | 1.56 (1.02-2.09) | 1.06E-01 | 1.87 (1.47-2.64) | 1.15E-01 | 1.23 (0.43-2.02) | 6.13E-01 |
| rs12465024 | C.G | 0.30 | 0.61 | 0.68 (0.39-0.97) | 8.44E-03 | 0.84 (0.63-1.27) | 4.32E-01 | 0.72 (0.29-1.14) | 1.23E-01 |
| rs112587259 | A.C | 0.11 | 0.55 | 0.33 (0.13-3.46) | **3.07E-06** | 0.44 (0.09-1.13) | 2.04E-02 | 0.47 (0.17-1.12) | 2.28E-02 |
| rs2422436 | A.G | 0.27 | 0.64 | 0.75 (0.47-1.04) | 5.52E-02 | 0.90 (0.68-1.32) | 6.20E-01 | 0.75 (0.33-1.18) | 1.92E-01 |
| rs58342673 | C.T | 0.30 | 0.6 | 0.68 (0.39-0.97) | 8.49E-03 | 0.84 (0.62-1.27) | 4.30E-01 | 0.71 (0.29-1.14) | 1.22E-01 |
| rs10209083 | G.A | 0.42 | 0.33 | 0.72 (0.36-1.08) | 7.83E-02 | 0.88 (0.61-1.41) | 6.32E-01 | 0.74 (0.21-1.26) | 2.50E-01 |
| rs62140432 | G.A | 0.30 | 0.55 | 0.75 (0.45-1.05) | 6.09E-02 | 0.92 (0.70-1.36) | 7.11E-01 | 0.75 (0.31-1.19) | 2.04E-01 |
| rs11893423 | A.G | 0.27 | 0.63 | 0.75 (0.47-1.04) | 5.55E-02 | 0.90 (0.68-1.32) | 6.20E-01 | 0.75 (0.33-1.18) | 1.93E-01 |
| rs6730986 | T.C | 0.27 | 0.63 | 0.75 (0.47-1.04) | 5.53E-02 | 0.90 (0.68-1.32) | 6.20E-01 | 0.75 (0.33-1.18) | 1.93E-01 |
| rs17753423 | A.G | 0.30 | 0.38 | 1.24 (0.90-1.15) | 2.23E-01 | 0.79 (0.51-1.32) | 3.73E-01 | 1.15 (0.64-1.66) | 5.93E-01 |
| rs2052261 | A.G | 0.27 | 0.63 | 0.75 (0.47-1.04) | 5.54E-02 | 0.90 (0.68-1.32) | 6.21E-01 | 0.75 (0.33-1.18) | 1.93E-01 |
| rs10208485 | G.A | 0.11 | 0.35 | 1.57 (1.02-2.12) | 1.06E-01 | 1.89 (1.49-2.68) | 1.14E-01 | 1.23 (0.42-2.05) | 6.12E-01 |
| rs10185243 | T.G | 0.29 | 0.56 | 0.64 (0.34-0.05) | 4.50E-03 | 0.81 (0.59-1.26) | 3.66E-01 | 0.69 (0.25-1.14) | 1.04E-01 |
| rs7568994 | T.G | 0.11 | 0.35 | 1.57 (1.02-2.11) | 1.06E-01 | 1.88 (1.48-2.67) | 1.14E-01 | 1.23 (0.43-2.04) | 6.11E-01 |
| rs12713532 | C.G | 0.27 | 0.62 | 0.75 (0.46-1.05) | 5.84E-02 | 0.90 (0.68-1.33) | 6.31E-01 | 0.75 (0.32-1.18) | 1.96E-01 |
| rs3732096 | T.C | 0.23 | 0.51 | 0.65 (0.31-0.99) | 1.21E-02 | 0.80 (0.55-1.30) | 3.91E-01 | 0.68 (0.19-1.17) | 1.22E-01 |
| rs2422437 | T.G | 0.23 | 0.51 | 0.65 (0.31-0.99) | 1.21E-02 | 0.80 (0.55-1.30) | 3.92E-01 | 0.68 (0.19-1.17) | 1.22E-01 |
| rs61758850 | C.T | 0.11 | 0.51 | 0.32 (0.15-3.58) | **3.27E-06** | 0.43 (0.07-1.15) | 2.12E-02 | 0.46 (0.21-1.13) | 2.30E-02 |
| rs6748462 | G.T | 0.26 | 0.47 | 0.56 (0.21-0.90) | **8.41E-04** | 0.74 (0.48-1.24) | 2.37E-01 | 0.63 (0.13-1.13) | 6.84E-02 |
