# Supplementary material for: Variants of CEP68 Gene Are Associated with Acute Urticaria/Angioedema Induced by Multiple Non-Steroidal Anti-Inflammatory Drugs
Source: PLoS One. 2014 Mar 11;9(3):e90966. doi: 10.1371/journal.pone.0090966 (PMC3949706; doi:10.1371/journal.pone.0090966)
Supplement: Table S6 — Results from the regression analyses on top associations at CEP68 and RAB1A for MNSAID-AU conditioning on the key SNP from CEP68 gene (rs1050675). (DOC) [file pone.0090966.s007.doc]

**Table S6. Results from the regression analyses on top associations at *CEP68* and *RAB1A* for MNSAID-AU conditioning on the key SNP from *CEP68* gene (rs1050675).**

| **SNP** | **Gene** | **p-value** |
| --- | --- | --- |
| rs6728523 | *CEP68* | 0.345 |
| rs2302647 | *CEP68* | 0.346 |
| rs2901749 | *CEP68* | 0.149 |
| rs2080385 | *CEP68* | 0.276 |
| rs75678687 | *CEP68* | 0.158 |
| rs79157909 | *CEP68* | 0.155 |
| rs7572857 | *CEP68* | 0.800 |
| rs17849707 | *CEP68* | 0.209 |
| rs12621608 | *CEP68* | 0.076 |
| rs76221156 | *CEP68* | 0.276 |
| rs1894874 | *CEP68* | 0.344 |
| rs113359765 | *CEP68* | 0.375 |
| rs6546125 | *CEP68* | 0.143 |
| rs78945874 | *CEP68* | 0.664 |
| rs1229 | *CEP68* | 0.996 |
| rs61758846 | *CEP68* | 0.872 |
| rs112029776 | *RAB1A* | 0.677 |
| rs113413623 | *RAB1A* | 0.787 |
| rs113506850 | *RAB1A* | 0.831 |
| rs76210337 | *RAB1A* | 0.707 |
| rs75263790 | *RAB1A* | 0.644 |
| rs9784068 | *RAB1A* | 0.808 |
| rs113467506 | *RAB1A* | 0.695 |
| rs75479685 | *RAB1A* | 0.692 |
| rs111245132 | *RAB1A* | 0.810 |
| rs111778391 | *RAB1A* | 0.827 |
| rs10176281 | *RAB1A* | 0.763 |
| rs113683435 | *RAB1A* | 0.833 |
| rs56725299 | *RAB1A* | 0.698 |
| rs17029863 | *RAB1A* | 0.689 |
| rs13409078 | *RAB1A* | 0.706 |
| rs1558658 | *RAB1A* | 0.722 |
| rs28392943 | *RAB1A* | 0.757 |
| rs13383649 | *RAB1A* | 0.736 |
| rs112587259 | *RAB1A* | 0.723 |
| rs61758850 | *RAB1A* | 0.665 |
